# How does the modality of delivering force feedback influence the performance and learning of surgical suturing skills? We don’t know, but we better find out! A review

**DOI:** 10.1007/s00464-022-09740-7

**Published:** 2022-10-27

**Authors:** Luca Oppici, Kim Grütters, Felix Bechtolsheim, Stefanie Speidel

**Affiliations:** 1 Psychology of Learning and Instruction, Department of Psychology, School of Science, Technische Universität Dresden, Zellescher Weg 17, 01069 Dresden, Germany; 2 Centre for Tactile Internet with Human-in-the-Loop (CeTI), Technische Universität Dresden, Dresden, Germany; 3 Norwegian School of Sport Sciences, Oslo, Norway; 4 Department of Visceral, Thoracic- and Vascular Surgery, University Hospital Carl Gustav Carus, Technische Universität Dresden, Dresden, Germany; 5 Division of Translational Surgical Oncology, National Center for Tumor Diseases Dresden, Dresden, Germany

**Keywords:** Knot tying, Needle inserting, Surgery, Surgical education, Surgical training

## Abstract

**Background:**

Force feedback is a critical element for performing and learning surgical suturing skill. Force feedback is impoverished or not present at all in non-open surgery (i.e., in simulation, laparoscopic, and robotic-assisted surgery), but it can be augmented using different modalities. This rapid, systematic review examines how the modality of delivering force feedback influences the performance and learning of surgical suturing skills.

**Methods:**

An electronic search was performed on PubMed/MEDLINE, Web of Science, and Embase databases to identify relevant articles. The results were synthesized using vote counting based on direction of effect.

**Results:**

A total of nine studies of medium-to-low quality were included. The synthesis of results suggests that the visual modality could be more beneficial than the tactile and auditory modalities in improving force control and that auditory and tactile modalities could be more beneficial than the visual modality in improving suturing performance. Results are mixed and unclear with regards to how modality affects the reduction of force magnitude and unclear when unimodal was compared to multimodal feedback. The studies have a general low level of evidence.

**Conclusion:**

The low number of studies with low methodological quality and low level of evidence (most were proof of concept) prevents us from drawing any meaningful conclusion and as such it is currently unknown whether and how force feedback modality influences surgical suturing skill. Speculatively, the visual modality may be more beneficial for improving the control of exerted force, while auditory and tactile modalities may be more effective in improving the overall suturing performance. We consider the issue of feedback modality to be highly relevant in this field, and we encourage future research to conduct further investigation integrating principles from learning psychology and neuroscience: identify feedback goal, context, and skill level and then design and compare feedback modalities accordingly.

Force feedback is a critical element for performing and learning surgical suturing skill [1, 2]. The suturing skill consists of driving and inserting a needle through the operated tissue, pulling the suture to close the wound, and tightly tying a knot to secure the suture. In this skill, perceiving the force exerted on the operated tissue allows an operator (surgeon or student) to properly handle instrument and tissue to successfully carry out the suturing procedure, tying a knot without leaking and avoiding damage to the tissue. We refer to force feedback as the feedback provided by a device/instrument concerning the use of forces and torques [3]. While being readily available to an operator in open surgery (primarily through the haptic sense), force feedback is impoverished or not present at all in non-open surgery (i.e., in simulation, laparoscopic, and robotic-assisted surgery). It is largely reduced in laparoscopic suturing due to presence of friction between the moving components inside the instrument and friction between trocar valves and instrument shaft, and it is absent in most robotic-assisted suturing (see the Senhance® surgical laparoscopic system for an exception). The lack of force feedback represents a critical issue for the performance of non-open surgical suturing and for the design of training interventions for medical students [4]. Surgeons themselves acknowledge the need for augmenting force feedback [5].

Recent technological developments allow for the provision of force feedback to an operator. Sensors placed on the instrument tip or below a tissue in simulation devices can capture the exerted force (e.g., see [6]). Force can then be delivered through augmented feedback and provide an operator with the sense of how much force they are applying on the suture and tissue. This in turn is expected to improve an operator’s control of force and consequently their suturing performance. Previous research has shown that augmenting force feedback is indeed effective in reducing the exerted force, improving an operator’s control of force, and there is some evidence that it can also improve suturing performance [3, 7, 8]. Importantly, force feedback can be delivered using different modalities: visual, auditory, tactile, and their combinations. For instance, pulling forces during knot tying can be delivered visually [9, 10], overlaying a force vector onto the laparoscopic video [11] or directly at the hand through tactile-based solutions [12]. Given the breadth of feedback modality options, the contexts in which feedback is needed (e.g., operating room or simulation-based training), and the goals for delivering feedback (i.e., assisting performance or promoting learning), it is highly relevant to examine the benefits and constraints of the different modalities of delivering force feedback.

The modality of delivering force feedback likely influences the performance and learning of surgical suturing skill. Considering that force in a suturing task is primarily controlled using tactile and kinesthetic sensory information [13, 14], one might think that the tactile modality is superior to the other modalities. However, the way a certain feedback modality influences learning and performance processes is complex and depends on its interaction with task complexity, a performer’s skill level, the goal of an intervention, and the environment in which feedback is expected to be used [15]. Research in motor skill learning provides evidence and some guidelines on how feedback modality operates in some specific situations. Importantly, skill performance and skill learning are two different constructs [16, 17] and a feedback modality may enhance performance but interfere with learning and vice versa. For example, the tactile modality has been shown to promote enhanced skill performance relative to the auditory modality in simple tasks and in novices, but the modality effect is reversed in complex tasks and in experts [18, 19]; auditory feedback is generally superior to visual in promoting learning, while visual might be better than auditory for performance, with different underlying neural activities [20, 21]. Furthermore, research in neuroscience and psychophysics is increasingly recognizing that feedback perception is a multimodal process, highlighting the benefits of multimodal over unimodal feedback [22, 23]. It is quite clear that choosing a feedback modality is a non-trivial and complex issue. Its effect cannot be generalized toward one modality or another (one size does not fit all), but modality effect has to be investigated in a specific skill (surgical suturing skill), population (students and surgeons), context (operating room and simulation-based training), and goal (assisting performance and promoting learning).

This review was conducted to examine how the modality of delivering force feedback influences the performance and learning of surgical suturing skills in laparoscopy. The results of this study can provide insights for the design of skill training interventions and assistance and can inform manufacturers of surgical-related instruments and trainers on the feedback modality to implement in their systems. For example, implementing haptics in a robotic simulator is expensive [24]. The results can inform whether such modality is essential and, if it is, it can justify the expense.

## Methods

The guidelines proposed by the 2020 Preferred Reporting Items for Systematic Reviews and Meta-Analyses (PRISMA 2020 [25]) and the guidelines from the Cochrane Rapid Reviews Methods Group [26] were followed. The research team, comprising experts in conducting systematic reviews (LO and SS) and surgery (FB), collectively designed the review process.

### Eligibility criteria

Inclusion criteria were set using a PICOS statement:

P (population): humans (students and surgeons).

I (intervention): force exerted on instrument–tissue interaction (pushing or pulling) is augmented during a surgery suturing task.

C (comparator): two or more feedback modalities of delivering force feedback are compared.

O (outcome): force parameters (main outcome) and surgical performance parameters (secondary outcome).

S (study design): experimental, quasi-experimental, and cross-over.

Studies that compared variations within a single feedback modality (e.g., different types of visual feedback) were not included. Furthermore, only peer-reviewed studies published in English were included.

### Information sources and search strategy

Three databases were searched to identify eligible studies: Pubmed, Web of Science, and Embase. The search was performed on the 14th of June 2022 and updated on the 4th of October 2022. Furthermore, the references of the studies included in the review were screened for identifying additional studies.

The search string comprised the following syntax: (force* OR kinesthe* OR pressure OR strength OR torque* OR tension) AND (feedback OR biofeedback OR substitution OR simulat* OR render* OR “virtual reality” OR “mixed reality” OR “augmented reality” OR “box trainer”) AND (haptic* OR tactile OR tactual OR vibro-tactile OR vibration OR audio OR auditory OR acoustic OR visual OR verbal OR modalit* OR type) AND (surgery OR surgic* OR laparoscop*) AND (sutur* OR knot-tying OR “knot tying” OR knot* OR “tissue manipulation” OR needle-driving OR “needle driving”).

### Selection process

**Figure 1:**
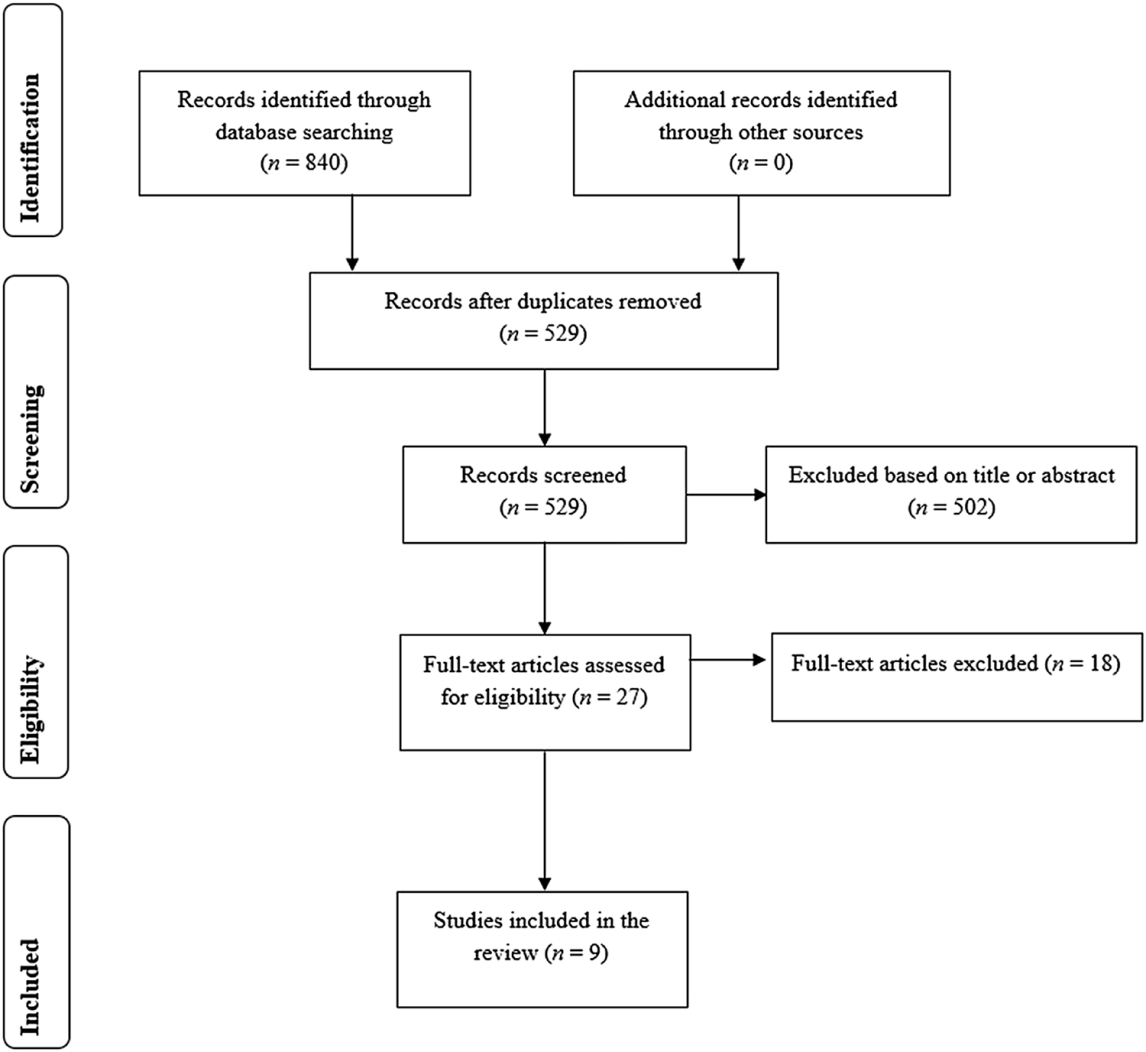
Flow diagram of the search and study selection process

The records identified through the database search were exported into Endnote X9 software (Clarivate) and duplicates were removed automatically first and then manually. One author (LO) screened titles and abstracts first and then the full texts. A second reviewer (KG) checked the excluded records, and any discrepancy was resolved in a closed meeting. If consensus was not reached, a third author (FB) was consulted (Fig. 1).

### Data collection process and data items

The following data were extracted from each study: general study information (author, year, study design), sample characteristics, study setting, suturing task and force parameter, feedback strategy, and outcome measures. One author (LO) extracted the data and a second author (KG) assessed data accuracy (Table 1).

**Table 1:** Summary of the studies characteristics

Study information	Sample characteristics	Procedure, suturing task and force measurement	Feedback strategies	Outcome measures
Al Fayyadh et al. [32] Randomized Controlled Trial (RCT)	*n* = 69 Age: 24 (21–35 years old) Intermediate: first- and second-year medical students with expert-level proficiency in the suturing task 5 groups (4 experimental and 1 control)	Procedure: 3 pre-test trials without feedback, practice trials to achieve proficiency or a maximum of 20 trials, 3 post-test trials without feedback Task: open surgical knot-tying task performed on a vessel ligation simulator Force: a sensor measured pushing and pulling force	Immediate auditory: real-time auditory feedback according to force magnitude. No sound < 0.3 N, 0.3 N < increasing sound pitch < 0.6 N, 0.6 N < high pitch and volume tone Delayed auditory: verbal feedback every 5th trial on the maximum exerted force Immediate visual: real-time continuous graph of the exerted force, displayed on a laptop Delayed visual: continuous recording of exerted force displayed every 5th trial Control: no feedback was provided during practice	Force: –Force magnitude: maximum absolute force, percentage of time exerting a force above 0.6 N Suturing performance: –Percentage of trials that leaked; total trial time; number of trials to achieve proficiency
Currie et al. [33] cross-over design	*n* = 12 Age: not specified Experts (*n* = 3) with over 500 robotics-assisted cardiac operations, and novices (*n* = 9) with no experience 8 feedback conditions	Procedure: each participant performed four trials in all experimental conditions in a randomized order Task: robotics-assisted mitral valve annuloplasty in a simulated test bed with a porcine mitral valve. Two tasks: pulling the suture a marked distance of 8 cm from the annuloplasty band (suturing) and tightening the suture knot (tying) Force: force sensor mounted below the porcine measuring applied force in x-, y-, and z-axis	Visual: force displayed in the form of a colored bar on the head-mounted display. Color and size change according to the force magnitude. Green < 4 N, 4 N < yellow < 6 N, and 6 N < red Direct haptic: force transmitted directly on the haptic wand of the master controller. Three levels of force provided: half, same, and double the intensity of the actual applied force Visual + haptic: combination of the previous conditions No feedback: no feedback provided	Force: –Force magnitude: maximum absolute force in both tasks
Howard and Szewczyk [34], cross-over design	*n* = 16 subjects Age: not specified Novices 7 feedback conditions	Procedure: each participant performed six unimodal feedback conditions and one of the multimodal conditions, in a randomized order. Three repetitions were performed in each condition Task: in a laparoscopic simulator, three suture-related wire pulling tasks were performed with the goal of reaching a defined pulling force, holding that force, and reaching that force three times changing the instrument orientation every time Force: a sensor measuring pulling force on the wire was placed on the simulation plate	Visual: real-time bar graph of force displayed on the endoscopic display Continuous vibrotactile: continuous feedback of linearly increasing amplitude Pulsed vibrotactile, fixed pulse length: vibration pulses of decreasing spacing proportional to the force applied Pulsed vibrotactile, varying pulse length, and interval: pulse length and spacing decreased proportional to force Pulsed vibrotactile, fixed pulse interval: pulse length decreased proportional to force Vibrotactile + visual: each of the previous vibrotactile condition was paired with the visual feedback No feedback: no feedback provided In all vibrotactile conditions, feedback was provided directly on participants’ hand through an actuator	Force: –Force control: precision in reaching a target force; speed of reaching the target force; quality of maintaining the force; force drift
Kitagawa et al. [35], cross-over design	*n* = 5 Age: not specified Intermediate: cardiac surgeons with 1 h of experience with robot-assisted surgery 5 feedback conditions	Procedure: 5 trials performed across 6 suture materials in each feedback condition. For a total of 30 repetitions in each condition, and 150 repetitions in total. First, the task was performed by hands and then with the robot Task: first throw of a surgical suture knot tied with hands and with a da Vinci robot Force: load cells tied to the suture measured pulling force	“Ideal feedback”: task performed by hand receiving natural intrinsic feedback Auditory: a single auditory tone was played when force reached the ideal feedback condition Visual: graph bar of the force was visually displayed Auditory + visual: combination of previous conditions No feedback: no feedback provided	Force: –Force magnitude: mean force –Force control: coefficient of variation of force
Mikic et al. [36], “case study” cross-over design	*n* = 4 Age: not specified Experts (*n* = 2) and novices (*n* = 2) 4 feedback conditions	Procedure: 5 trials were performed in each condition. Order was not randomized Task: in a robotics-assisted simulation, passing a suture through two wound edges followed by a double knot to secure the suture Force: a sensor measuring pulling force on the wire was placed on the simulation plate	Auditory: a continuous pitch was played on participants’ ears. The pitch amplitude is proportional to the force Visual: a continuous color was displayed at the side of the surgical environment. The color shade was proportional to the force Auditory + visual: combination of previous conditions No feedback: no feedback provided	Force: –Force magnitude: percentage of time above a threshold; maximum force
Talasaz et al. [37], cross-over design	*n* = 7 Age: not specified Expert (*n* = 1), intermediates (*n* = 3), and novices (*n* = 3) 3 feedback conditions	Procedure: 5 trials in each feedback conditions in a randomized order Task: secure the second throw of a surgical knot in a robotics-assisted simulation Force: strain gauges added to the cable shafts measuring forces at the tip of the instruments	Visual: force displayed visually using three colors: green < 4 N, 4 N < yellow < 6 N, 6 N < red Direct haptic: force was directly provided on the haptic wand No feedback: no feedback provided	Force: –Force magnitude: absolute pulling force –Force control: consistency of force Suturing performance: –Knot quality (whether it leaked or not); instrument–tissue collision force; number of tissue–instrument hits; total trial time
Talasaz et al. [38], cross-over design	*n* = 16 Age: not specified Experts (*n* = 3) and novices (*n* = 13) 3 feedback conditions	Procedure: 5 trials in each feedback conditions in a randomized order Task: two tasks in a robotics-assisted simulation –Insert the needle by puncturing into a simulated tissue and grasp the tip of the needle to pull it out of the tissue –secure the second throw of surgical knots Force: a sensor was placed under the simulated tissue	Visual: force displayed visually using three colors: green < 4 N, 4 N < yellow < 6 N, and 6 N < red Direct haptic: force was directly provided on the haptic wand No feedback: no feedback provided	Force: –Force magnitude: absolute pulling force Suturing performance: –Knot quality (whether it leaked or not); instrument–tissue collision force; number of tissue–instrument hits; total trial time
Tavakoli et al. [39], cross-over design	*n* = 7 Age: 24 to 34 years old Novices 4 feedback conditions	Procedure: 10 trials with 3 suture materials in each condition, 30 trials per condition Task: insert a needle in a simulated tissue using a tele-operated system Force: rotating force was measured	Visual Direct haptic, low gain Direct, high gain Visual + haptic high gain Details of the feedback strategies are not provided	Force: –Force magnitude: peak and average force
Tavakoli et al. [40], cross-over design	*n* = 8 Age: 24 to 34 years old Novices 4 feedback conditions	Procedure: 10 trials with 4 suture materials in each condition, 40 trials per condition. Order was randomized Task: insert a needle in a simulated tissue using a tele-operated system Force: rotating force was measured	Visual Direct haptic Visual + haptic No feedback Details of the feedback strategies are not provided	Force: –Force magnitude: peak and average force

### Study risk of bias assessment

The Risk of Bias tools developed by the Cochrane group were used for the risk of bias assessment [27]. Considering the design of the included studies (one RCT and cross-over trials), the RoB 2.0 tool was used. This tool is primarily designed for RCTs and is composed of five bias domains: (1) randomization process, (2) deviations from the intended interventions, (3) missing outcome data, (4) measurement of the outcome, and (5) selection of the reported results. A modified version of the tool, which contains the five domains just described and an additional domain for bias arising from period and carryover effects, was used for cross-over trials. Signaling questions help the assessment of the potential bias in each domain. Each domain has three possible outcomes—low, some concerns, and high. An overall outcome, corresponding to the highest risk across domains was calculated for each study (i.e., if the risk was *some concerns* in one domain only but *low* in all other domains, the overall risk was *some concerns*). The results of the risk of bias assessment are presented using the traffic light system: green (low), yellow (some concerns), and red (high) (see Table 2). One author (LO) assessed the risk of bias using the excel spreadsheet available at https://www.riskofbias.info/welcome and a second author (KG) assessed data accuracy.

**Table 2:**
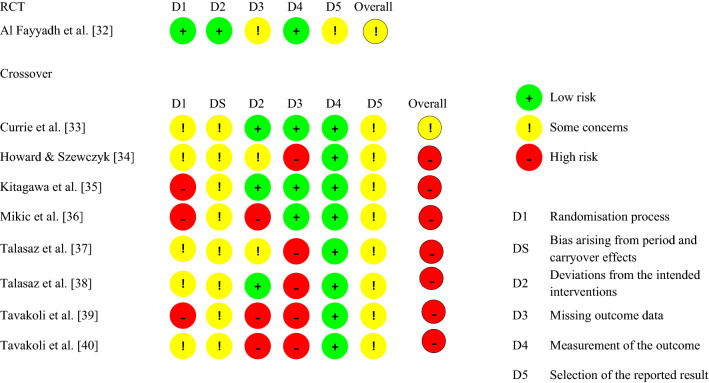
Results of the Risk of Bias assessment for the included studies, evaluated with the RoB 2.0 tool (Color figure online)

### Synthesis methods

Due to inconsistency of the effect measures and data reported across studies, it was not possible to compute a meta-analysis, and we decided to synthesize results using vote counting based on direction of effect. Despite some limitations (i.e., does not provide an estimate of effect magnitude, does not account for different study sizes, and it is less powerful than combining *p* values), this synthesis is a valid method for estimating the overall direction of an effect [28]. The direction of effect was calculated in each study from descriptive statistics or graphs on the outcome of interest. In the context of comparing two feedback modalities, three direction outcomes were possible: in favor of one modality, in favor of the other modality in the comparison, or no change/mixed effects/conflicting findings [29]. An effect was classified in favor of one modality or the other when at least 70% of the analyzed outcomes reported a similar direction, while it was classified no change/mixed effects/conflicting findings if otherwise. Statistical significance was not considered for assessing the direction [30]. Studies were first grouped by the compared modality(ies) (Table 3) and then they were grouped by the examined task(s) (Table 4). It would have been highly relevant to group studies in learning-focused and performance-focused studies; however, only one study evaluated skill learning (see overview of study characteristics for more details) and it was not possible to perform such grouping. In both cases, participants with different skill levels were combined, as studies did not report any modality–skill level interaction effect. Some studies had different intervention conditions (e.g., compared unimodal and multimodal modalities) and appear in the synthesis multiple times with different results. The effect direction was synthesized using the sign test: nonparametric test computed to examine the probability of observing the obtained direction of effect if the null hypothesis (i.e., equal number in one direction and the other) were true [31]. The number of results in favor of one modality or another was counted, and the *p*-value was computed using the sign and binomial test on GraphPad website (https://www.graphpad.com/quickcalcs/binomial1/).

**Table 3:**
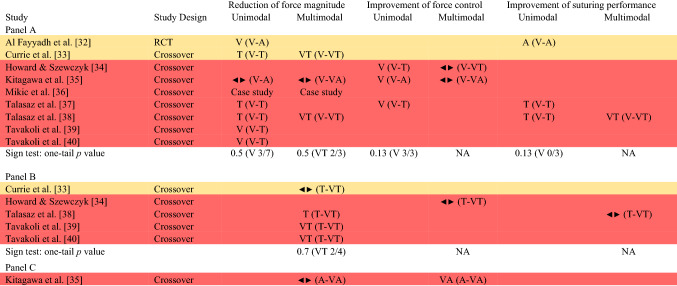
Direction of effect plot with combined tasks and skill levels (Color figure online)

Panel A includes studies that compared visual with either auditory or tactile modality (unimodal), and visual with either visual + auditory or visual + tactile (multimodal)

Panel B includes studies that compared tactile with tactile + visual

Panel C includes a study that compared auditory with auditory + visual

Study design: *RCT* Randomized Controlled Trial, and Crossover

Effect direction in favor of: V = visual, A = auditory, T = tactile, VA = visual + auditory, VT = visual + tactile; sideways arrow ◄► = no change/mixed effects/conflicting findings. The comparison each effect refers to is specified in parenthesis

Study quality: denoted by row color: amber = some concerns; red = high risk of bias

**Table 4:**
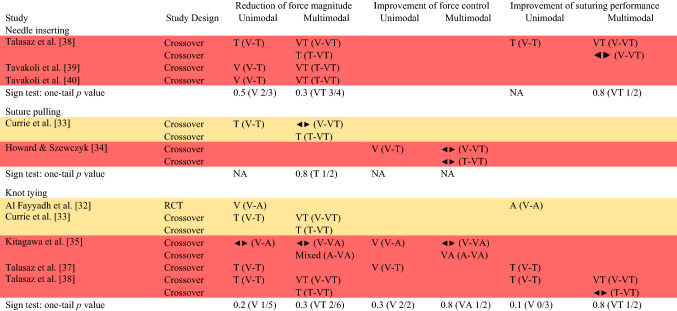
Direction of effect plot with studies clustered in the task examined (Color figure online)

Study design: *RCT* Randomized Controlled Trial, and Crossover

Effect direction in favor of: V = visual, A = auditory, T = tactile, VA = visual + auditory, VT = visual + tactile; sideways arrow ◄► = no change/mixed effects/conflicting findings. The comparison each effect refers to is specified in parenthesis

Study quality: denoted by row color: amber = some concerns; red = high risk of bias

## Results

### Search

The search resulted in a total of 840 studies (234 in Pubmed, 242 in Web of Science, and 364 in Embase). After duplicates removal, 529 studies were screened and 502 were excluded based on their title or abstract. Twenty-seven full texts were then screened and 18 studies were excluded because did not meet the inclusion criteria. As such, a total of nine studies were included in the review (Fig. 1).

### Overview of study characteristics

A detailed description of the studies is presented in Table 1. One study was a Randomized Controlled Trial (RCT) [32], while the other eight studies adopted a cross-over design, whereby each participant was exposed to different feedback conditions [33–40]. Only the RCT, comprised a learning phase and a post-test without feedback, evaluated the effect of feedback on skill learning, while the other studies evaluated the effect of feedback on skill performance. A total of 141 participants were recruited with a median sample size of 8. Three studies recruited novices only, two studies recruited participants with an intermediate skill level, and the other studies recruited a mix of novices, intermediates and experts. The number of practice trials performed in each experimental condition (or group) varied between 3 and 30, with a median of 5. One study used an entire surgical suturing task, while the other studies isolated the single components (i.e., inserting the needle, pulling the suture, and tying the knot). Except for two studies that adopted a laparoscopic or open simulator, robotics-assisted and tele-operated surgical systems were employed. With regards to the modality of delivering force feedback, all studies compared visual with either tactile or auditory modality, and most studies also compared unimodal (visual, tactile, auditory) with multimodal feedback (combination of visual with tactile or auditory). Lastly, outcome measures included force magnitude in all studies, force control, and suturing performance in some studies.

### Study risk of bias

The methodological quality of the studies is predominantly low: two studies had ‘some concerns’ [32, 33], and the others had a high risk of bias [34–40]. The randomization procedure in most cases was poorly conducted or reported, and it was not reported whether the randomization of feedback conditions was counter-balanced in cross-over studies [33–40]. Furthermore, in some cases it was not reported whether all participants and outcomes were included in the analysis [34, 37–40], and how the analysis estimated the effect of assignment to intervention. Lastly, none of the studies reported how data analysis was planned before data collection and eventually adapted during/after data collection.

### Synthesis of results

The effect direction plot of studies grouped by the examined modality is presented in Table 3.

#### Visual vs tactile or auditory

**Reduction of force magnitude** The direction of effect is mixed.

**Improvement of force control** The effect is in the direction of the visual modality (*p* = 0.13), with three studies out of three in favor of this modality.

**Improvement of suturing performance** The effect is in the direction of the modality other than the visual (*p* = 0.13). Two studies are in favor of the tactile modality, one in favor of the auditory modality, and none in favor of the visual modality.

#### Visual vs multimodal (visual + tactile or auditory)

**Reduction of force magnitude** The direction of effect is mixed.

**Improvement of force control** The direction of effect is mixed.

**Improvement of suturing performance** The effect of the only study included is in the direction of a multimodal strategy (visual + tactile).

#### Tactile vs multimodal (tactile + visual)

**Reduction of force magnitude** The direction of effect is mixed.

**Improvement of force control** The effect of the only study included is mixed/unclear.

**Improvement of suturing performance** The effect of the only study included is mixed/unclear.

The effect direction plot of studies grouped by the examined task is presented in Table 4.

#### Needle inserting task

**Reduction of force magnitude** The direction of effect is mixed both for unimodal and multimodal comparisons.

**Improvement of suturing performance** The direction of effect is mixed both for unimodal and multimodal comparisons.

#### Suture pulling task

**Reduction of force magnitude** The direction of effect is mixed both for unimodal and multimodal comparisons.

**Improvement of force control** The direction of effect is mixed both for unimodal and multimodal comparisons.

#### Knot-tying task

**Reduction of force magnitude** The direction of effect is mixed both for unimodal and multimodal comparisons.

**Improvement of force control** The direction of effect is in the direction of the visual modality in unimodal comparisons, and the direction is mixed in the multimodal comparisons.

**Improvement of suturing performance** The direction of effect is in the direction of the modality other than visual in the unimodal comparisons, and the direction is mixed in the multimodal comparisons.

#### Level of evidence

Overall, the level of evidence is low due to the predominant use of a study design (cross-over) with a lower level of evidence than RCTs and a predominant high risk of bias.

## Discussion

This review examined how delivery modality influences the effect of force feedback on surgical suturing skills. In other words, what are the benefits and constraints of different force feedback modalities (e.g., visual and tactile) on suturing performance and learning? A limited number of studies (*n* = 9) with a relatively high risk of bias and low level of evidence were included in the review. The results of synthesis suggests that the visual modality could be more beneficial than the tactile and auditory modalities in improving force control and that auditory and tactile modalities could be more beneficial than the visual modality in improving suturing performance. Results are mixed and unclear with regards to how modality affects the reduction of force magnitude. Results show mixed and unclear effects across all the outcomes considered when unimodal (e.g., visual and tactile) was compared with multimodal (e.g., visual plus tactile) force feedback. Similarly, in the knot-tying task, the visual modality could be more beneficial than the other modalities in improving force control, and auditory and tactile modalities could be more beneficial than the visual modality in improving performance. Results across the other tasks and outcomes were mixed and unclear. These results were synthesized combining participants with different levels of expertise (e.g., novices and experts), as the design and analysis of the included studies did not allow to separate the effects according to skill level.

Considerable resources are increasingly invested in developing and refining interventions for training medical student’s surgical skills and for assisting surgeons’ performance in the operating room [41]. Force feedback is widely recognized as a key component of such interventions [1, 2]. Quite surprisingly, this review showed that a low number of studies with low methodological quality examined how force feedback modality influences learning and performance of surgical suturing skill. This can have two main underlying reasons: the feedback modality has no relevant effect on skill development or its potential effect is not known in this specific medical field. Considering that research in psychology and neuroscience indicates that different feedback modalities (e.g., visual and tactile) influence motor skills differently [15] with distinct neural pathways [20], we consider the second explanation to be the most plausible. In support of this, we can observe on a meta-level that the effect direction is mixed due to a diversity of study design with confounding variables—and not due to mixed, unclear direction in each study. This might suggest that indeed modality influences force feedback effectiveness (e.g., there was an overall trend for the visual modality to improve force control and visual and tactile modalities for improving task performance), but the studies’ low methodological quality and low level of evidence prevent us from drawing any meaningful conclusion. As such, rather than speculating on potential feedback modality effect using the weak collected evidence, we discuss how relevant would it be to properly conduct research on this issue. Starting from the limitations of the included studies, we provide a guideline for future research regarding this topic.

### Impediments in previous studies and suggestions for future research

The studies included in this review present several limitations. Previous studies were often poorly designed from a methodological perspective. The sample size and consequently statistical power were low. None of the studies used a power analysis to inform the required sample size for testing the anticipated modality effect. Randomization of conditions was poorly conducted and reported, with potential order effect and unbalance across conditions. The plan for data analysis and the actual data analysis was poorly reported, e.g., it was not clear whether all outcome measures were analyzed in all participants. These are the main methodological limitations that future research should address and improve.

On a theoretical level, the influence and collaboration of learning psychology and neuroscience were either not noticeable or at least not discussed. The main limitation was the lack of a principled approach underlying the design and comparison of feedback modalities. Predominantly, feedback modalities were selected on convenience and technical considerations, as if modality effect could generalize to all tasks, contexts, and individuals. We suggest future research to consider these critical aspects of feedback design.

A study should define the goal of a feedback strategy, i.e., promoting students’ skill learning or assisting surgeons’ skill performance in the operating room. In the former, feedback dependency could represent an issue and feedback modality should be selected accordingly. Feedback dependency occurs when a learner regulates their movement on the feedback information—not on intrinsic information—and performance improvement vanishes when feedback is removed [42]. This clearly interferes with learning. On the other hand, feedback dependency is less of an issue in a strategy for assisting surgeons’ performance. If a feedback strategy is always present in the operating room, a surgeon can rely on this augmented information to regulate their movement. Feedback modality can then be designed accordingly. Previous research in motor learning has shown that the auditory modality is generally more beneficial for learning (reduced feedback dependency) and the visual modality for performance [19, 43]. Future research should examine whether this trend occurs also in surgical suturing skill. Furthermore, another promising research avenue—rarely investigated, but highly relevant for suturing skill—is the influence of tactile modality on feedback dependency. A good starting point for researching this issue is knowing the distinction between performance and learning [16, 17].

Another important aspect related to the goal of a feedback strategy is the context in which feedback is implemented or the context toward which a trained skill is intended to transfer. A feedback strategy should be designed considering the characteristics of the target task and environment. For example, visual and tactile modalities may be preferable to an auditory modality for assisting surgeons in laparoscopic surgery whereby environmental noise would prevent the perception of acoustic feedback. On the other hand, auditory feedback may be suitable for assisting surgeons in robotic-assisted surgery in a non-noisy, quite environment. Furthermore, future research should systematically examine how skill level interacts with feedback modality. The studies included in this review did recruit participants with different levels of expertise, but in a confused, non-systematic manner (e.g., different samples and inappropriate data analysis). It was also not stated the rationale for recruiting different skill levels and the impact their level may have on feedback modality effect. Previous research indicates that this is a highly relevant factor and warrants a more thoughtful approach [18, 19]. For example, a criticality for feedback effectiveness is a performer’s mapping of the provided feedback onto their movement, i.e., knowing what the feedback means and how to change movement accordingly. Novices tend to prefer the tactile or visual modality, while experts seem to benefit more from the auditory modality [15]. Another critical aspect to consider is the complexity of the task and movement involved in performing such task and the related complexity of information to provide to a learner/performer (for a detailed overview of this issue see [44, 45]). Tasks and movement with large degrees of freedom (e.g., knot tying) requires feedback that somewhat covers those degrees of freedom and the modality(ies) can play a significant role here. A combination of modalities may be used to provide different information or a single modality may be designed to cover different aspects of a movement. Lastly, the included studies did not control for feedback content as confounding factor [45]. Descriptive and prescriptive feedback have a differential effect on skill. The visual modality can be either descriptive or prescriptive, while auditory and tactile are primarily prescriptive. Auditory and tactile are provided in reference to a threshold (to activate or to smooth). The study design should keep feedback content consistent across modalities for a proper evaluation of modality effect.

This review presents some limitations worth mentioning. Studies were required to be published in English to be included, which may have excluded studies published in other languages. Further, evidence was synthesized using vote counting based on direction of effect, which is a valid method but less precise than a meta-analysis.

In summary, the studies included in this review presented a series of methodological and theoretical limitations, from a motor skill learning perspective. We have provided suggestions for overcoming these limitations. It is crucial for future research to consider the multifactorial interaction of feedback modality with a learner, task, and context. We encourage researchers to consider our suggestions and delineate a detailed plan when investigating the effect of feedback modality on surgical skill. Furthermore, researchers should specify which conditions (goal, context, and skill level) an observed effect can be generalized to. If these issues are not addressed, a study, at best, can be a proof of concept, without providing meaningful results on skill learning and performance. We acknowledge that the technical elements for a feedback modality strategy are complex and require technical expertise (engineers in various fields), but we also emphasize the necessity of including in the research team researchers with expertise in study design and human factors (learning psychology and neuroscience). In this context, an interdisciplinary team is highly recommended [46].

## Conclusion

Augmenting force feedback is a key issue in surgical suturing skill, especially in non-open surgery (laparoscopy, robotics assisted, and teleoperation) whereby naturally occurring feedback is impoverished. Different modalities of providing force feedback exist, with potentially different effects on skill learning and performance. This review identified nine studies that examined this issue, comparing different modalities. The low number of studies with low methodological quality and low level of evidence (most were proof of concept) prevent us from drawing any meaningful conclusion and as such it is currently not known whether and how force feedback modality influences surgical suturing skill. We consider the issue of feedback modality to be highly relevant in this field, and we encourage future research to conduct further investigation integrating principles from learning psychology and neuroscience: identify feedback goal, context, and skill level and then design and compare feedback modality accordingly.
